# Secreted Amyloid Precursor Protein β and Secreted Amyloid Precursor Protein α Induce Axon Outgrowth In Vitro through Egr1 Signaling Pathway

**DOI:** 10.1371/journal.pone.0016301

**Published:** 2011-01-27

**Authors:** Stéphanie Chasseigneaux, Levent Dinc, Christiane Rose, Claude Chabret, Fanny Coulpier, Piotr Topilko, Gweltas Mauger, Bernadette Allinquant

**Affiliations:** 1 INSERM U894, Paris, France; 2 Université Paris-Descartes, Paris, France; 3 Developmental Biology Section, INSERM U1024, CNRS UMR 8197, Institut de Biologie de l'Ecole Normale Supérieure, Paris, France; 4 Plate-Forme Transcriptome INSERM U1024, CNRS UMR 8197, Institut de Biologie de l'Ecole Normale Supérieure, Paris, France; Biological Research Center of the Hungarian Academy of Science, Hungary

## Abstract

**Background:**

sAPPα released after α secretase cleavage of Amyloid Precursor Protein (APP) has several functions including the stimulation of neurite outgrowth although detailed morphometric analysis has not been done. Two domains involved in this function have been described and are present in sAPPβ released at the first step of amyloid peptide cleavage, raising the possibility that sAPPβ could also stimulate neurite outgrowth. We investigated the morphological effects of sAPPα and sAPPβ on primary neurons and identified a key signaling event required for the changes observed.

**Methodology/Principal Findings:**

Final concentrations of 50 to 150 nM bacterial recombinant sAPPα or sAPPβ added to primary neuronal cultures after 1 day in vitro decreased cell adhesion 24 hours later and primary dendrite length 96 hours later. 150 nM sAPPα and sAPPβ induced a similar increase of axon outgrowth, although this increase was already significant at 100 nM sAPPα. These morphological changes induced by sAPPs were also observed when added to differentiated neurons at 5 days in vitro. Real time PCR and immunocytochemistry showed that sAPPα and sAPPβ stimulated Egr1 expression downstream of MAPK/ERK activation. Furthermore, in primary neurons from Egr1 −/− mice, sAPPs affected dendritic length but did not induce any increase of axon length.

**Conclusion/Significance:**

sAPPα and sAPPβ decrease cell adhesion and increase axon elongation. These morphological changes are similar to what has been observed in response to heparan sulfate. The sAPPα/sAPPβ stimulated increase in axon growth requires Egr1 signaling. These data suggest that sAPPβ is not deleterious per se. Since sAPPβ and sAPPα are present in the embryonic brain, these two APP metabolites might play a role in axon outgrowth during development and in response to brain damage.

## Introduction

In addition to being a key molecule in Alzheimer's disease, Amyloid Precursor Protein (APP) and its metabolites play important roles during brain development [1]. APP appears at embryonic (E) day 9.5 in mouse when the first neurons have started to differentiate [2]. APP cleavage by alpha secretase generates secreted APP (sAPPα), which is present during brain development [3]. sAPPα stimulates the proliferation of neural stem cells from embryonic rat neocortex and from adult mouse brain [4], [5]. sAPPα has neurotrophic and neuroprotective properties, and recently, it was shown to increase LTP and spatial memory [1], [6], [7].

Specific domains of sAPPα have been identified that contribute to neuroprotection and others to the stimulation of neurite outgrowth in vitro [6]. However, little is known about the effects of sAPPβ, generated by beta secretase cleavage, and which shares the same sequence as the sAPPα except for the last 16 C-terminal amino acids. Cleavage by β secretase occurs upstream to γ secretase cleavage and generates the amyloid peptide, which can form soluble neurotoxic oligomers and is the main component of extracellular amyloid deposits in Alzheimer pathology. After cleavage of the amyloid peptide the APP C-terminal domain is released and enters the nucleus where it can affect gene expression [8]. APP cleavage and signaling also occur during brain embryogenesis and seem to be necessary for normal brain development [9]–[11]. A recent report showed that in peripheral neurons deprived growth factor and that undergo apoptosis, β cleavage releases sAPPβ, which binds to DR6 inducing neurodegeneration [12]. Compared to sAPPα, sAPPβ is 100-fold less potent in protecting hippocampal neurons against excitotoxicity, amyloid toxicity and glucose deprivation [13]. Although sAPPα stimulates neurite outgrowth, a detailed morphometric analysis has never been done. Two domains located between residues 96–110 and 319–335 in sAPPα are reported that contribute to neurite outgrowth. The former region is also a binding site for heparan sulfate proteoglycans (HSPG) [14], [15]. Both of these domains are present in the sAPPβ, suggesting that sAPPβ could also stimulate neurite outgrowth.

The signaling pathways involved in sAPPα neuroprotection have been characterized. Less well known are the signaling pathways involved in sAPPα neurotrophic properties. Recently, we and others have shown that mitogen activated protein kinase (MAPK)/extracellular signal-regulated (ERK) pathway is activated during neurite outgrowth of neural stem cell derived neurons or primary neurons in response to sAPPα [16]–[18].

Here, we examined whether sAPPβ also stimulated neurite outgrowth and compared this with sAPPα. We observed that both induce similar and specific effects on axon outgrowth and that their effects require Egr1 signaling.

## Methods

### sAPP-Fc production

A plasmid encoding human sAPPα (695 amino acid form) fused to the Fc fragment of human IgG was transfected into Cos-7 cells and sAPP-Fc purified from the conditioned medium on a protein A-sepharose column using standard procedures [5].

### Cloning, expression and purification of recombinant sAPPα and sAPPβ

We first generated a DNA fragment harbouring the coding sequence for sAPPα and sAPPβ by PCR amplification of a plasmid encoding for human APP695. For both proteins the forward primer (Eurofins MWG Operon) was 5′-ACTGTCGACTATGCTGCCCGGTTTGGCA-3′ containing Sal1 restriction site. The reverse primers were 5′-CAGCGGCCGCTTTTTGATGATGAACTTC-3′ for sAPPα and 5′-CAGCGGCCGCCATCTTCACTTCAGAGA-3′ for sAPPβ, both primers containing Not1 restriction site. The amplified DNA were cloned into pGEX-6P-2 (GE Healthcare), containing a PreScission Protease sequence upstream to the Sal1 restriction site for cleaving the GST tag from the fused protein. The generated plasmids were sequenced.

The plasmids were transformed into *E.coli* strain BL21pLysS (Invitrogen). The transformed cells were grown in 25 ml of YT broth medium containing 100 µg/ml ampicilline at 37°C for 16 h then diluted 1/10 in the same medium and grown to an absorbance of 0.3 at 600 nm. Proteins encoded by the plasmids were induced by addition of isopropyl-β-D-thiogalactopyranoside (IPTG) to a final concentration of 0.1 mM and incubated for an additional 3 h at 20°C. The bacteria were then collected by centrifugation, resuspended in 5 ml of phosphate buffer saline (PBS) containing 0.2 mg/ml lysozyme and 5 mM dithiothreitol, and lysed with a sonicator 1 min at 4°C. Triton X100 1% was added and the mixture was stirred gently for 2 h at 4°C and then centrifuged 15 min at 14,000 g at 4°C. The supernatants were added to a glutathione-sepharose column equilibrated with binding buffer. After washings, proteins were eluted from glutathione-sepharose beads with PreScission Protease (GE Healthcare) at 4°C during 24 h, according to the manufacturer's instructions. Recombinant proteins were analyzed by SDS-PAGE for their respective MW and checked by immunoblot using the N-terminal APP antibody (MAB 348, Chemicon International) recognizing both proteins, while the C-terminal specific sAPPα antibody (6E10, Signet) did not recognize the sAPPβ.

### Ethics statement

All animal procedures followed the French and European Union regulations. The protocol of animal euthanasia was performed according to French government ethical laws decree 86/609 and approved by the local Ethics Committee (Direction départementale des services vétérinaires de Paris, service de la protection et santé animales et de la protection de l'environnement) permit number: 75–1003.

### Primary cortical neurons

Primary neuronal cultures from cortex were performed from E16 mouse embryos from Swiss strain mice. Briefly, dissociated cells were plated (15×10^3^ neurons/well for morphometric analysis, and 40×10^3^ neurons/well for adhesion assays) in 16.2 mm diameter plastic wells coated with 1.5 µg/ml polyornithin. For immunocytochemical studies neurons were plated on glass coverslips coated with 15 µg/ml polyornithine at a density of 10^5^ neurons/well. Neurons were grown in a define medium free of serum and supplemented with hormones, proteins, and salts as previously described [19]. Final concentrations of 50 to 150 nM sAPP-Fc, sAPPα or sAPPβ were added to neurons after 1 day in vitro (DIV) for the different tests. In some experiments final concentration of 150 nM sAPPs was added to the neurons after 5DIV.

### Morphometric analysis

After addition of sAPPs on 1DIV neurons, cells were fixed 4 days later with 4% paraformaldehyde for 40 min at room temperature. In experiments where neurons were treated for 1 h with 5 µM of ERK1,2 inhibitor (UO126, Sigma), the cells were fixed 2 days later as above. Then neurons were washed twice in PBS and incubated with anti-β tubulin (1/5000, Sigma) overnight at 4°C. After two washes in PBS, neurons were incubated with a biotinylated anti-mouse antibody, and immunoreactivity was visualized using an Elite ABC kit (Vector Laboratories) and 3,3′-diaminobenzidin as the chromogen. For each condition of each experiment, about 60 neurons were digitally acquired and morphologically analyzed with NeuronJ software. The length of the longest neurite, the length of all primary dendrites and the number of primary dendrites for each neuron were evaluated independently by two different experimenters (SC and LD). Statistical analysis was performed using ANOVA and post-hoc Scheffe's test (StatView). The data (Mean ± S.E.M.) are presented in supplemental tables and the corresponding graphs are presented in figures. Significance is shown in the figure legends.

### Adhesion assays

For adhesion assays, neurons grown in presence of sAPPs for 24 h were agitated for 10 min at 250 revolutions/min at room temperature, the medium containing detached cells was removed and the remaining neurons adhering to the bottom of the well were fixed with 2.5% glutaraldehyde in PBS for 30 min, rinsed in PBS and stained with Toluidine blue [20]. For each condition of each experiment, neurons on a diameter of 3 wells were counted independently by two different experimenters (SC and LD).

### Immunocytochemistry

For Egr1 immunostaining, neurons after 1DIV were incubated or not for 1 h with 5 µM of ERK1,2 inhibitor (UO126, Sigma), and treated 2 h with sAPPs. Neurons were then fixed as above, rinsed in PBS, and non-specific binding sites saturated for 1 h at 37°C in PBS with 3% bovine serum albumin (BSA) and 0.2% Triton X100. The primary antibody for Egr1 (C19, Santa Cruz) was diluted 1/2000 in PBS with 1% BSA and 0.2% Triton X100 and incubated for 16 h at 4°C. After 3 rinses in PBS, coverslips were incubated with anti-rabbit antibody coupled to cyanine-3 (1/400, Jackson ImmunoResearch) diluted in PBS 1% BSA for 1 h at 37°C. After 3 washes, coverslips were mounted in medium containing 4′6′-diamidino-2-phenylindole (DAPI) (Vector Laboratories) and neurons examined by epifluorescence microscopy. For each condition, about 1,400 neurons were counted on 3 coverslips independently by two experimenters (SC and LD).

For microtubule-associated protein 2 (MAP2) and Tau labelling, neurons were fixed as above, rinsed in PBS, and saturated for 1 h at 37°C with PBS containing 10% fetal calf serum (FCS) and 0.05% Tween 20. The primary monoclonal antibody for MAP2 (1/500, Chemicon International) and polyclonal affinity purified anti-Tau (1/2000, AbCam) were diluted in saturation buffer and incubated for 1 h at 37°C. After rinses, coverslips were incubated with anti-mouse antibody conjugated to Alexa-488 (1/200, Jackson ImmunoResearch) and cyanine-3 anti-rabbit antibody (1/500, Jackson ImmunoResearch) for 1 h at 37°C. After washes in PBS, coverslips were mounted in a medium containing DAPI and neurons examined by confocal laser scanning microscopy.

### Real time PCR for quantification of mRNA levels

Neurons after 1DIV were incubated or not for 1 h with 5 µM of ERK1,2 inhibitor (UO126, Sigma) then for 50 min with sAPPs. The medium was removed, the cells were washed once in PBS and then collected by scraping. Total RNA was extracted using the RNeasy mini kit and treated with DNaseI (Qiagen). cDNAs were obtained from total RNA samples using a High Capacity cDNA Reverse Transcription kit (Applied Biosystems). Briefly, 20 µl of reaction mixture containing 100 ng of total RNA, 1X reverse transcription buffer, 1 mM of each dNTP, 1X reverse transcription random primer and 50U of MultiScribe reverse transcriptase were incubated at 25°C for 10 min followed by incubation at 37°C for 2 h. The reaction was stopped by heating at 85°C for 5 sec. Real time PCR was performed using an Abi Prism 7000 (Applied Biosystems) and the Absolute SYBR Green ROX Mix (ThermoScientific). 25 ng of cDNAs were mixed with 0.07 µM of each primer, 1X SYBR Green Mix and PCR-grade water to a final volume of 25 µl. The cycling conditions for all primers (Egr1 forward: 5′-GGGGAGCCGAGCGAACAAC-3′, Egr1 reverse: 5′-TGATGGGAGGCAACCGAGTC-3′, β actin forward: 5′-CCCCAACTTGATGTATGAAG-3′ and β actin reverse: 5′-GCACTTTTATTGGTCTCAAG-3′) were 50°C for 2 min, then 95°C for 15 min followed by 40 cycles consisting of two steps, 15 sec at 95°C and 1 min at 60°C. The PCR program was completed by a melting temperature analysis consisting of 15 sec at 95°C, 20 sec at 60°C and then steps through which temperature ranged from 60 to 95°C. Amplification plots were produced to calculate the threshold cycle (Ct) and standard curves of Ct versus log cDNA dilution were generated for both target and reference genes. Since all reactions were done in triplicate, the average Ct was used. Relative quantification was calculated using the 2^-ΔΔCt^ method. Relative expressions of the genes were assessed in the sAPPs (sAPP-Fc, sAPPα and sAPPβ) and UO126 treated neurons compared to the corresponding untreated cells.

### Egr1 mutant mice

Embryos E16 from Egr1 heterozygous pregnant mice (C57Bl/6J background) were genotyped using the tail of each embryo as described [21]. Briefly, a mixture of three oligonucleotides was used for PCR amplification: a common 5′-primer mapping in the Egr1 coding sequence (5′-GAGTGTGCCCTCAGTAGCTT-3′) and two different 3′-primers, mapping in the Egr1 coding sequence (5′-GGTGCTCATAGGGTTGTTCGCT-3′) and in the lacZ-coding sequence (5′-AACGACTGTCCTGGCCGTAACC-3′), respectively. PCR amplification was performed with Taq polymerase (ABGene) under the conditions recommended by the supplier and involved 30 cycles consisting of 1 min at 92°C, 2 min at 60°C, and 2 min at 72°C.

At the same time, the cortex of each embryo was dissected, dissociated and distributed individually for primary neuronal cultures in plastic wells of 16.2 mm diameter at a density 15×10^3^ neurons/well. Then sAPPs were added the next day and 4DIV later, the cells were fixed, stained for β tubulin and morphometric analyses were performed.

## Results

### sAPPα and sAPPβ induce a decrease of cell adhesion and an increase of the axon length in vitro independently of the post-translational modifications

**Figure 1:**
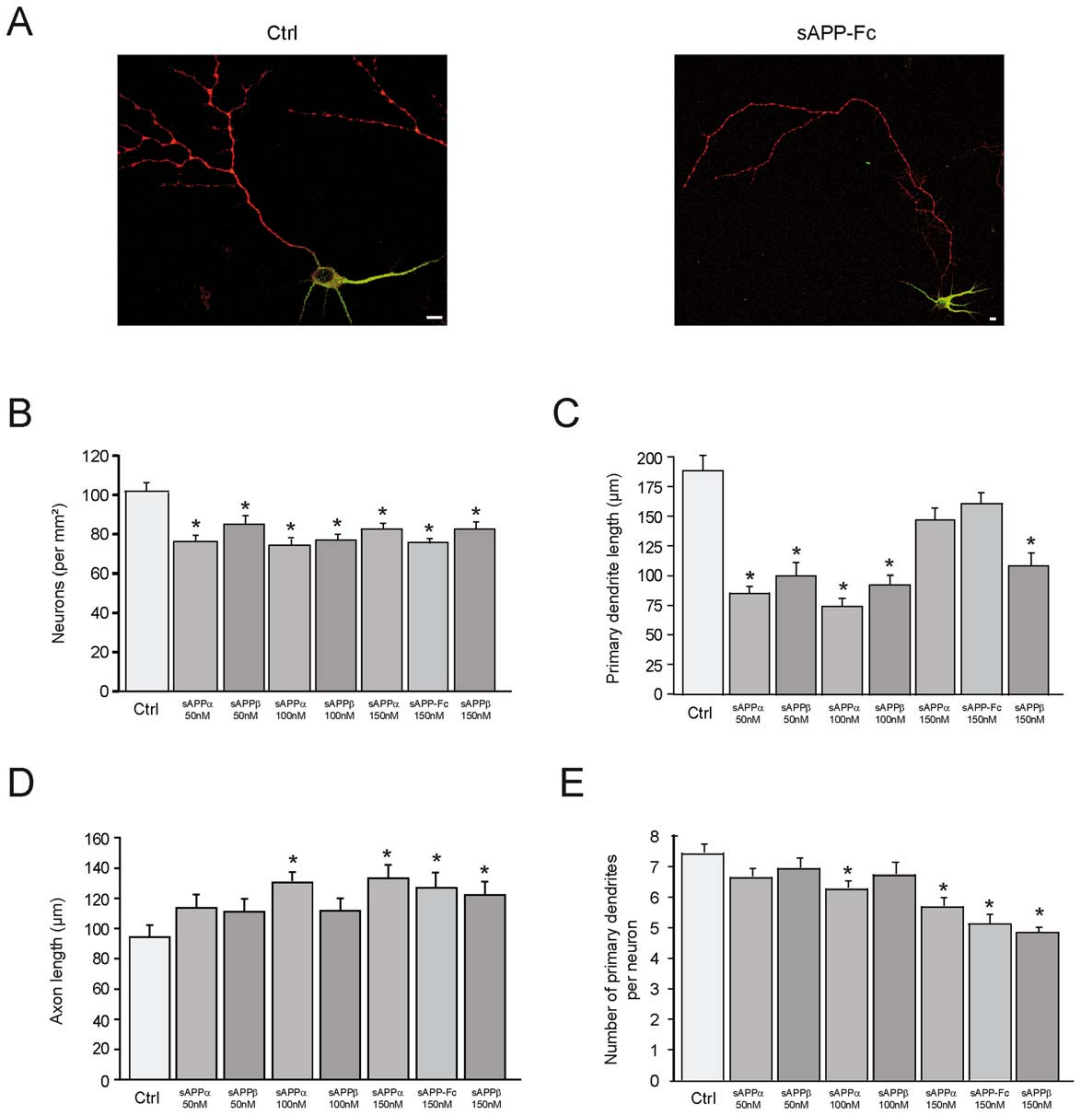
Changes in cell adhesion and in neuronal morphology induced by sAPPα, sAPPβ and sAPP-Fc added to neurons after 1 DIV. (**A**) 5DIV neurons were immunostained for MAP2 (green) and Tau (red). Representative confocal merged images show that the longest neurite is stained for Tau, an axonal marker, in control (Ctrl) neurons or after addition of sAPP-Fc to 1DIV neurons. Scale Bar: 10 µm. (**B**) Adhesion is significantly reduced after sAPPα or sAPPβ (50 to 150 nM). sAPPα and sAPPβ were added to primary neurons after 1DIV and adhesion assays were performed 24 hours later. (50 nM: control (Ctrl) vs sAPPα: p<0.001, control vs sAPPβ: p<0.04; 100 nM: control vs sAPPα: p<0.0001, control vs sAPPβ: p<0.0005; 150 nM: control vs sAPPα: p<0.003, control vs sAPP-Fc: p<0.005, control vs sAPPβ: p<0.01). (**C–E**) Morphometric analysis 96 hours after the addition of sAPPs (50 to 150 nM) to 1DIV primary neurons. (C) At 50 and 100 nM sAPPα and sAPPβ induce a significant decrease of primary dendrite length while at 150 nM, the decrease in primary dendrite length is still significant for sAPPβ only (50 nM: control (Ctrl) vs sAPPα: p<0.0001, control vs sAPPβ: p<0.0001; 100 nM: control vs sAPPα: p<0.0001, control vs sAPPβ: p<0.0001; 150nM: control vs sAPPα: NS, control vs sAPP-Fc: NS, sAPPα vs sAPP-Fc: NS, control vs sAPPβ: p<0.0001). (D) At 150 nM sAPPα, sAPP-Fc and sAPPβ induce an increase of axon elongation (150 nM: control (Ctrl) vs sAPPα: p<0.005; control vs sAPP-Fc: p<0.002; control vs sAPPβ: p<0.003; sAPPα vs sAPPβ: NS; sAPPα vs sAPP-Fc: NS; sAPPβ vs sAPP-Fc: NS). At 100 nM sAPPα was already significant (100 nM: control vs sAPPα: p<0.034). (E) shows a significant decrease of the number of primary dendrites for sAPPα, sAPP-Fc and sAPPβ at 150 nM and starting at 100 nM for sAPPα (100 nM: control (Ctrl) vs sAPPα: p<0.035; 150 nM: control vs sAPPα: p<0.0001, control vs sAPP-Fc: p<0.0001; control vs sAPPβ: p<0.0001). The data presented are the mean of 3 independent experiments. Statistical analysis was done with ANOVA and Scheffe's post-hoc test.

We previously produced a recombinant protein of human sAPPα 695 fused to the Fc domain of human immunoglobulin in the conditioned medium of transfected Cos-7 cells [5]. This recombinant protein bears all the post-translational modifications observed in eukaryotic cells. Here, we generated two other recombinant proteins for sAPPα and sAPPβ produced in bacteria. The recombinant proteins (50 to 150 nM final concentrations) were added to cultures of embryonic primary cortical neurons after 1 day in vitro (DIV) and 96 hours later, the cells were fixed and stained for β tubulin for morphometric analysis. Using double staining for MAP2 and Tau, we verified that in 5DIV neurons the longest neurite was the axon in control neurons as well as in neurons after addition of any of the 3 recombinant proteins to 1DIV (Fig. 1A).

**Figure 2:**
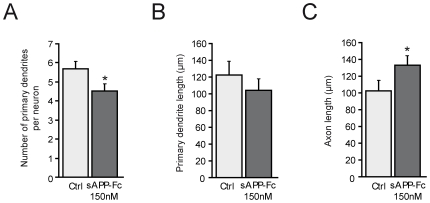
Changes in neuronal morphology after 150 nM sAPP-Fc added to 5DIV neurons. Morphometric analysis 96 hours after the addition of 150 nM sAPP-Fc to 5DIV neurons shows that: (**A**) sAPP-Fc induces a significant decrease of primary dendrite number (control (Ctrl) vs sAPP-Fc: p<0.0276). (**B**) sAPP-Fc does not modify primary dendrite length (control vs sAPP-Fc: NS). (**C**) sAPP-Fc induces an increase of axon elongation (control vs sAPP-Fc: p<0.0297). The data in A-C are the mean of 3 independent experiments. Statistical analysis was done with ANOVA and Scheffe's post-hoc test.

Since substrate adhesion influences the development of axon and dendrites in vitro, we first analyzed the effect of sAPPα and sAPPβ on the adhesion to 1DIV primary neurons [19], [20], [22]. Adhesion tests revealed a significant decrease in cell numbers 24 hours after the addition of sAPPα and sAPPβ and this decrease was similar for final concentrations of 50 nM to 150 nM (Table S1 and Fig. 1B). Morphometric analysis 96 hours after addition of sAPPα and sAPPβ to 1DIV neurons showed a significant decrease of primary dendrite length at concentrations of 50 to 100 nM for sAPPα and to 150 nM for sAPPβ (Table S1 and Fig. 1C). At 150 nM sAPPα and sAPPβ showed a similar and significant increase of axon outgrowth (Table S1 and Fig. 1D). This increase was also significant at 100 nM for sAPPα (Table S1 and Fig. 1D). The increase of axon outgrowth induced by sAPPα and sAPPβ was associated with a significant decrease of the number of primary dendrites (Table S1 and Fig. 1E). Similar changes in cell adhesion and morphology were observed with sAPP-Fc suggesting that post-translational modifications are not required for the effects on neurite outgrowth induced by sAPPα (Table S1 and Fig. 1B–E). When 150 nM sAPP-Fc was added to cultures already 5DIV when the differentiation is complete and then examined 96 hours later, we observed similar morphological changes in the number of primary dendrites, primary dendrite length and axonal elongation that we observed when the protein was added to 1DIV neurons (Table S1 and Fig. 2). Furthermore, the increase in axonal outgrowth after the addition of sAPP-Fc to 5DIV neurons reached the same length (Mean ±S.E.M.: Ctrl: 102.8±11.5 µm; sAPP-Fc: 132.6±10.2 µm) as that when the protein was added to 1DIV neurons (Ctrl: 90±6.8 µm; sAPP-Fc: 127±9.2 µm), which appears to be the maximum axonal length response to this amount of sAPP-Fc.

### Egr1 is induced by sAPPs

**Figure 3:**
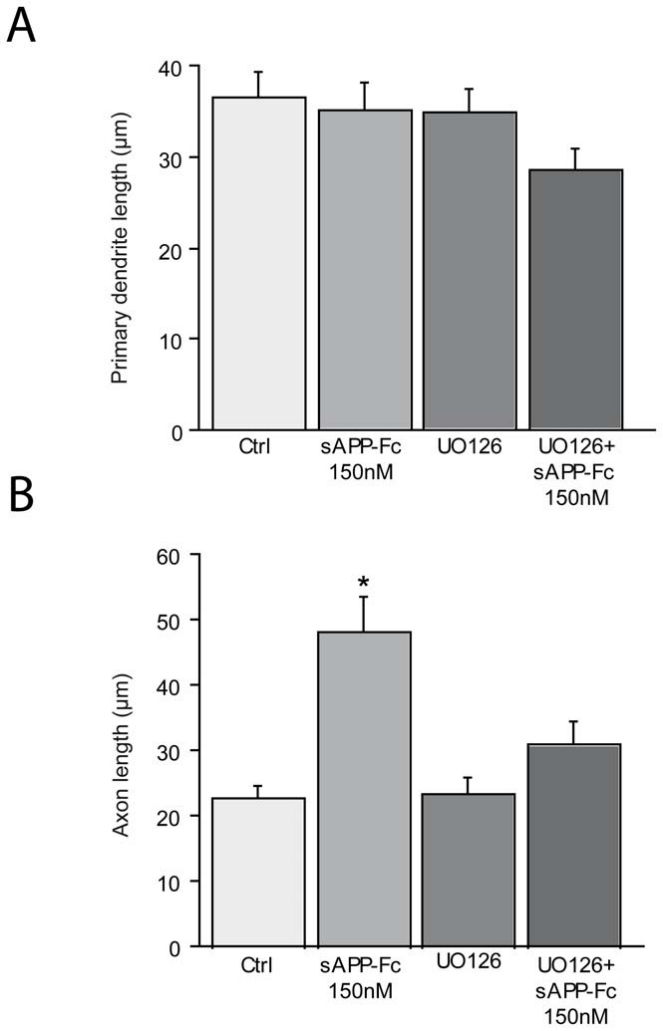
UO126 inhibits changes in neuronal morphology after 150 nM sAPP-Fc added to 1DIV neurons. UO126 was added to neurons after 1DIV 1 hour before the addition of 150 nM sAPP-Fc. Morphometric analysis was performed 48 hours later. (**A**) UO126 does not induce any change in primary dendrite length by itself nor in combination with sAPP-Fc (control (Ctrl) vs sAPP-Fc: NS; control vs UO126: NS; control vs UO126+ sAPP-Fc: NS). (**B**) UO126 1 hour before the addition of sAPP-Fc significantly reduces the growth of axons induced by sAPP-Fc (control vs sAPP-Fc: p<0.0001; control vs UO126: NS; control vs UO126+ sAPP-Fc: NS). The data presented in A and B are the mean of 3 independent experiments. Statistical analysis was done with ANOVA and Scheffe's post-hoc test.

**Figure 4:**
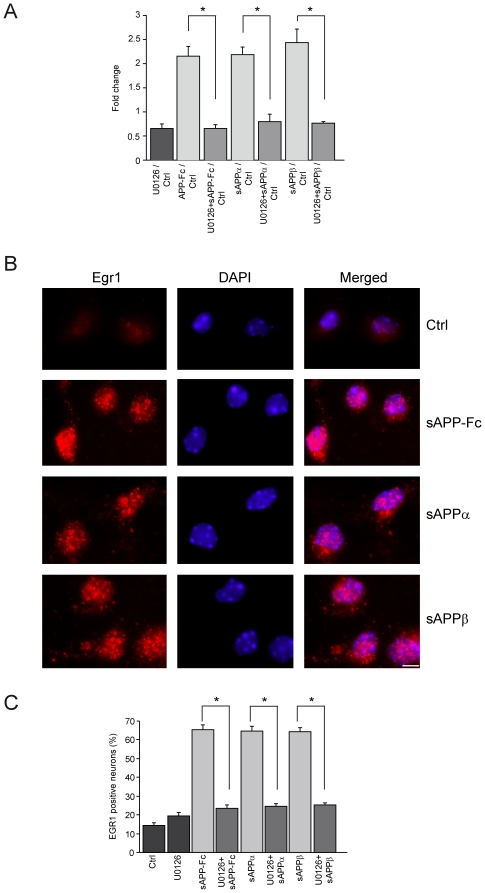
sAPPα, sAPPβ and sAPP-Fc induce Egr1 expression. (**A**) Real time PCR for each recombinant protein added to 1DIV neurons for 50 minutes shows a 2-fold increased expression of Egr1 compared to controls (Ctrl). The addition of UO126 1 hour before the addition of sAPPs significantly decreases Egr1 expression (sAPP-Fc/control after UO126 vs sAPP-Fc/control: p<0.0001; sAPPα/control after UO126 vs sAPPα/control: p<0.0001; sAPPβ/control after UO126 vs sAPPβ/control: p<0.0015). (**B**) Egr1 immunofluorescence 2 hours after the addition of sAPPs. The nuclear labelling by DAPI is presented and the merged images show an increase of Egr1 protein in the nucleus after sAPPs. Scale Bar: 10 µm. (**C**) Quantification of the % of nuclear Egr1 positive neurons 2 hours after addition of sAPPs only and after addition of UO126 1 hour before sAPPs (control (Ctrl) vs sAPP-Fc: p<0.0001; control vs sAPPα: p<0.0001; control vs sAPPβ: p<0.0001, control vs UO126: NS; sAPP-Fc vs UO126+ sAPP-Fc: p<0.0001; control vs UO126+ sAPPα: p<0.0001; control vs UO126+ sAPPβ: p<0.0001). For each condition, about 1,400 neurons were counted on 3 coverslips. The data presented in A and C are the mean of 3 independent experiments. Statistical analysis was done with ANOVA and Scheffe's post-hoc test.

We have recently shown that MAPK/ERK is activated early by sAPPα in differentiating neurons [17]. Here, we tested whether UO126, a specific inhibitor of ERK, abolished the sAPPs-induced increase in axonal elongation (Table S1 and Fig. 3). The genes downstream of ERK that act directly on neurite outgrowth have not been determined. Egr1 is one possible candidate, since in PC12 cells NGF induces Egr1 downstream of ERK and EGR1 is required in the differentiation of neuroblastoma cells [23], [24]. We used real time PCR to examine Egr1 transcript levels at short times after the addition of sAPPs and observed a maximum response at 50 min. We observed a similar significant increase of Egr1 transcript with the 3 recombinant proteins (Fig. 4A). This action on Egr1 was abolished when UO126 was added 1 hour before sAPPs, suggesting that Egr1 acts downstream of MAPK (Fig. 4A). We confirmed this at the protein level using an antibody specific for Egr1. We detected a significant increase in the number of intensively EGR1 labeled neurons 2 hours after the addition of sAPPs. This increase was blocked by the addition of UO126 1 hour before sAPPs (Fig. 4B–C).

### Egr1 induced by sAPPs is directly involved in the axon outgrowth in response to sAPPs

**Figure 5:**
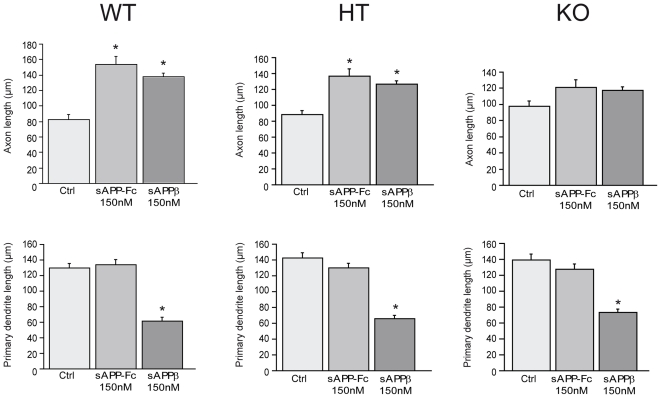
The primary cortical neurons from E16 homozygous knock out embryos for Egr1 do not respond to sAPPs. 150 nM sAPP-Fc or sAPPβ were added on 1DIV primary neurons from Egr1 wild type (WT), heterozygous (HT) and homozygous (KO) embryos. Morphometric analysis was performed 96 hours later. Upper row: A significant increase of axonal outgrowth is observed with sAPP-Fc and sAPPβ for neurons from WT and HT embryos but not from KO neurons (WT: control (Ctrl) vs sAPP-Fc: p<0.0001, control vs sAPPβ: p<0.0001; HT: control vs sAPP-Fc: p<0.001, control vs sAPPβ: p<0.0001; KO: control vs sAPP-Fc: NS, control vs sAPPβ: NS). Lower row: Primary dendrite length is significantly decreased for sAPPβ in the 3 genotypes (WT: control vs sAPP-Fc: NS, control vs sAPPβ: p<0.0001; HT: control vs APP-Fc: NS, control vs sAPPβ: p<0.0001; KO: control vs APP-Fc: NS, control vs sAPPβ: p<0.0001). The data presented are the mean of 3 independent experiments. Statistical analysis was done with ANOVA and Scheffe's post-hoc test.

To examine whether Egr1 was involved in sAPPs-induced axon outgrowth, we used embryonic primary neurons from Egr1 mutant mice. In each litter we genotyped each embryo and plated neurons from wild type, heterozygous and homozygous animals separately for 1DIV before adding sAPPs. 96 hours later the cells were fixed and morphological analyses performed. We observed a significant increase in axon length after the addition of sAPP-Fc and sAPPβ to cultures of neurons from wild type and heterozygous embryos but no increase in cultures of neurons from homozygous mutant animals (Table S2 and Fig. 5). Interestingly, sAPPβ induced a significant decrease of dendrite length in primary neurons from all genotypes suggesting that the effects on dendrite reduction and axon outgrowth may be regulated by different pathways. 150 nM sAPP-Fc had no effect on dendrite length regardless of the genotype and this is similar to what we observed in primary neurons from wild type mice with another genetic background (Fig. 1, Fig. 5). These data suggest that Egr1 is directly required in the sAPPs-induced axon outgrowth and not in dendrite outgrowth responses.

## Discussion

### sAPPβ and sAPPα induce similar morphological changes in primary neurons

sAPPβ induces an increase in axon outgrowth that is similar to that caused by sAPPα. This effect on axon outgrowth is independent of post-translational modifications on the proteins. However sAPPα was more efficient as the effect on axonal elongation was already present at a lower concentration, suggesting that the 16 amino-acids lacking in the C-terminal part of sAPPβ bears some information for accelerating the axonal outgrowth.

The increase in axonal outgrowth induced by sAPPα and sAPPβ is likely linked to the decrease in cell adhesion. A relationship between axonal outgrowth and decreased adhesion has previously been described and has been attributed to changes in the cytoskeleton [19], [20], [22]. Large amounts of fasciculated microtubules in the axon may increase axon cytoplasmic viscosity and require less adhesion to the substrate. Conversely, dendrite elongation is related to a strong cellular adhesion and to low viscosity [19], [20], [22]. In our studies the decrease in adhesion is the first event after addition of sAPPs and is observed 24 hours later and at all concentrations of 50 to 150 nM. Then, the decrease in dendrite outgrowth occurs 72 hours later, starting at concentrations of 50 nM, suggesting that the decrease in cell adhesion either stops or prevents the growth of dendrites, as has been observed by others using heparan sulfate on primary neurons [19], [20], [22]. The decrease in adhesion leads to the simultaneous elongation of the axon and retraction of some primary dendrites starting at 100 nM for sAPPα and 150 nM for sAPPβ. Retraction of primary dendrites may be necessary for axonal outgrowth in that the soma will have a lower adhesion and this will allow the axon to grow. Although the decrease in cell adhesion and in dendrite outgrowth were equivalent at 50 and 100 nM, a higher concentration of sAPPα was necessary for increasing axonal outgrowth, suggesting that a reduction of adhesion is not sufficient for promoting axon outgrowth. At 150 nM, the highest concentration used, some dendrite outgrowth occurs with both sAPPα and sAPP-Fc as if the decrease in dendrite outgrowth observed at a lower concentration was lost. The increase of dendrite outgrowth after 150 nM sAPPα and sAPP-Fc indicates that the maximal axonal outgrowth is reached and that dendrite outgrowth can occur. Here, we cannot exclude that the same can be observed for sAPPβ at a higher concentration. Interestingly, this increase of dendrite outgrowth at 150 nM reflects what occurs during brain embryogenesis where the neurons generated in the germinal zone migrate to their normal position extending their axons and elaborating their dendritic tree once the axon terminals have reached their target [22].

Two domains in sAPPα one located between residues 319–335 and the other between residues 96–110 have been shown to convey neurotrophic effects [13], [14]. The latter is a heparin binding domain that interacts with HSPGs [14]. The effects of sAPPα and sAPPβ on axonal outgrowth that we observe here are associated with a decrease in cell adhesion that is similar to what has been described for heparan sulfate on primary neurons [19], [20]. Indeed, previous reports have shown that heparan sulfate added to neurons in vitro after plating induce a specific increase of axonal outgrowth associated to a decrease of cell adhesion and a clear absence of dendrites, while dermatan sulfate increases the cell adhesion and stimulates mostly dendrite outgrowth [20]. When full length APP is bound to the substratum, HSPGs appear to be necessary to stimulate neurite outgrowth and the physiological responses observed varies with HSPG type [25]–[27]. Although we observed that sAPPs can interact with some HSPGs in the conditioned medium of 1DIV primary neurons (not shown), we do not yet know if this interaction accounts for the effects of sAPPs that we report here.

The 16 amino acids lacking in the sequence of sAPPβ have been shown to be a heparin binding domain as well [13]. This domain appears not to be necessary for increasing axon outgrowth, but it is required for neuroprotection and LTP [13], [7]. The addition of sAPPs was performed to 1DIV neurons, a time where the differentiation machinery starts, and to 5DIV when the neurons are already differentiated. In that case, the addition of 150 nM sAPP-Fc induces an increase of axonal outgrowth that reaches the same length as that after addition to 1DIV, and which appears to be the maximum axonal growth response. The action of sAPP-Fc on neurons after 5DIV suggests that in cases of neurite retraction in vivo following insults to neurons, the sAPPs might be therapeutically useful for recovery of synaptic connections. Indeed, sAPPα is able to protect against ischemic brain injury [28]. The mechanisms of sAPPα action including a putative receptor are not known. sAPPα interacts with the cell surface of 3 different cell lines [29] and we previously observed such binding with neuroblasts derived from the subventricular zone and grown as neurospheres in which sAPPα stimulates proliferation [5]. In primary neurons we observed sAPP-Fc binding at the cell surface (not shown) but we have yet to identify molecular partners for this binding. We recently reported that the N-methyl-D-aspartic acid receptor is required for the sAPPα induced neurite outgrowth from neural stem cell-derived neurons [17]. sAPPα can compete with APP at the cell surface for integrin β1 interaction resulting in an increase in neurite outgrowth via integrin signaling [30]. Whether this is also the case for sAPPβ has not yet been determined. Since the increase in axon elongation is first observed at 100 nM for sAPPα, it is possible that the 16 amino acids lacking in the sAPPβ play a role in this physiological effect and/or be responsible for a conformational change reducing the effects of the two upstream domains (319–335 and 96–110 amino-acids) to a putative membrane receptor for this physiological response.

These data and the recent report showing that β-amyloid monomers are neuroprotective suggest that cleavage by β secretase is not deleterious per se [31]. It does not preclude that sAPPβ can act in a death signal when a partner like DR6 is highly expressed [12].

### Egr1 is a key step in the sAPPs-induced axonal elongation

We previously showed that MAPK/ERK is activated early in sAPPα induced neurite outgrowth [17]. Egr1 activation has already been shown to be downstream of ERK1/2. In PC12 cells, NGF stimulates ERK inducing Egr1 expression [23], [24]. In this case, Egr1 mediates the induction of p35 activating Cdk5, which is necessary for neurite-like outgrowth. Here we observed an increase in Egr1 transcript 50 min after addition of the recombinant sAPPα, sAPPβ or sAPP-Fc. The contribution of Egr1 is downstream of MAPK/ERK pathway since it is inhibited by UO126. The MAPK/ERK pathway is not involved in changes in primary dendrites since we observed no effects on control or on sAPP-Fc-treated neurons in the presence of UO126. When sAPPs were added to primary neurons from embryonic mice homozygous for Egr1, an increase in axon outgrowth was not observed as in neurons from embryonic wild type mice from the same litter, while the effects on dendrite outgrowth were observed with all genotypes. These data suggest that Egr1 signaling occurs at the beginning of axon elongation induced by sAPPs and not through an effect on dendrite outgrowth. We did not observe any significant morphological differences in the responses of embryonic neurons from wild type and heterozygous mutant mice, suggesting that one functional allele of Egr1 is sufficient to promote axonal elongation induced by sAPPs. The fact that neurons from mice homozygous for Egr1 are unable to respond to sAPPs with an increase in axon outgrowth suggests that Egr1 is a key component in the signaling pathway and that this step is not compensated by other genes. Interestingly, a previous report that used the same mutant mice showed a deficit in late LTP and long-term memory and established that Egr1 is required for these functions [32]. Indeed, sAPPα has been shown to increase LTP and spatial memory [7]. It is interesting to speculate about whether the expression of Egr1 that is necessary for LTP is dependent of sAPPα. If this is the case, it will be important to understand why sAPPβ stimulates axon elongation through Egr1 while it does not act in LTP [7]. Whether Egr1 is also a key step in the neuroprotective or neuroproliferation functions of sAPPα remains to be determined.

## Supporting Information

Table S1 **Presentations of parameters investigated in** **Figures 1** **,** **2** **, and** **3** **.** In Figure 1, sAPPs was added to 1DIV neurons at concentrations of 50 to 150 nM; cell adhesion and morphometric analysis was performed 24 hours and 96 hours later, respectively. In Figure 2, sAPP-Fc at 150 nM was added to 5DIV neurons and morphometric analysis was performed 96 hours later. In figure 3, UO126 was added 1 hour before sAPP-Fc at 150 nM on neurons after 1DIV and morphometric analysis was performed 48 hours later. The data are expressed as the Mean ± S.E.M. The levels of significance are indicated by a star and described in the corresponding legends of Figs. 1, 2, and 3. The primary neurons were from mouse embryonic cortex of E16 Swiss strain mice.
(DOC)

Table S2 **Presentations of parameters investigated in** **Figure 5** **.** In Figure 5, 150 nM sAPP-Fc or sAPPβ were added to 1DIV embryonic cortical neurons from wild type (WT), heterozygous (HT), homozygous (KO) neurons from Egr1 (C57Bl/6J background) mutant mice. Morphometric analysis was performed 96 hours later. The data are expressed as Mean ± S.E.M. The levels of significance are indicated by a star and detailed in the legend of Fig. 5.
(DOC)

## References

[1] Gralle M, Ferreira ST (2007). Structure and functions of the human amyloid precursor protein: the whole is more than the sum of its parts.. Prog Neurobiol.

[2] Salbaum JM, Ruddle FH (1994). Embryonic expression pattern of amyloid protein precursor suggests a role in differentiation of specific subsets of neurons.. J Exp Zool.

[3] Loffler J, Huber G (1992). Beta-amyloid precursor protein isoforms in various rat brain regions and during brain development.. J Neurochem.

[4] Ohsawa I, Takamura C, Morimoto T, Ishiguro M, Kohsaka S (1999). Amino-terminal region of secreted form of amyloid precursor protein stimulates proliferation of neural stem cells.. Eur J Neurosci.

[5] Caille I, Allinquant B, Dupont E, Bouillot C, Langer A (2004). Soluble form of amyloid precursor protein regulates proliferation of progenitors in the adult subventricular zone.. Development.

[6] Mattson MP (1997). Cellular actions of beta-amyloid precursor protein and its soluble and fibrillogenic derivatives.. Physiol Rev.

[7] Taylor CJ, Ireland DR, Ballagh I, Bourne K, Marechal NM (2008). Endogenous secreted amyloid precursor protein-alpha regulates hippocampal NMDA receptor function, long-term potentiation and spatial memory.. Neurobiol Dis.

[8] Cao X, Sudhof TC (2001). A transcriptionally [correction of transcriptively] active complex of APP with Fe65 and histone acetyltransferase Tip60.. Science.

[9] Hartmann D, De Strooper B, Saftig P (1999). Presenilin-1 deficiency leads to loss of Cajal-Retzius neurons and cortical dysplasia similar to human type 2 lissencephaly.. Curr Biol.

[10] Herms J, Anliker B, Heber S, Ring S, Fuhrmann M (2004). Cortical dysplasia resembling human type 2 lissencephaly in mice lacking all three APP family members.. EMBO J.

[11] Guenette S, Chang Y, Hiesberger T, Richardson JA, Eckman CB (2006). Essential roles for the FE65 amyloid precursor protein-interacting proteins in brain development.. EMBO J.

[12] Nikolaev A, McLaughlin T, O'Leary DD, Tessier-Lavigne M (2009). APP binds DR6 to trigger axon pruning and neuron death via distinct caspases.. Nature.

[13] Furukawa K, Sopher BL, Rydel RE, Begley JG, Pham DG (1996). Increased activity-regulating and neuroprotective efficacy of alpha-secretase-derived secreted amyloid precursor protein conferred by a C-terminal heparin-binding domain.. J Neurochem.

[14] Small DH, Nurcombe V, Reed G, Clarris H, Moir R (1994). A heparin-binding domain in the amyloid protein precursor of Alzheimer's disease is involved in the regulation of neurite outgrowth.. J Neurosci.

[15] Ninomiya H, Roch JM, Jin LW, Saitoh T (1994). Secreted form of amyloid beta/A4 protein precursor (APP) binds to two distinct APP binding sites on rat B103 neuron-like cells through two different domains, but only one site is involved in neuritotropic activity.. J Neurochem.

[16] Greenberg SM, Qiu WQ, Selkoe DJ, Ben-Itzhak A, Kosik KS (1995). Amino-terminal region of the beta-amyloid precursor protein activates mitogen-activated protein kinase.. Neurosci Lett.

[17] Gakhar-Koppole N, Hundeshagen P, Mandl C, Weyer SW, Allinquant B (2008). Activity requires soluble amyloid precursor protein alpha to promote neurite outgrowth in neural stem cell-derived neurons via activation of the MAPK pathway.. Eur J Neurosci.

[18] Rohe M, Carlo AS, Breyhan H, Sporbert A, Militz D (2008). Sortilin-related receptor with A-type repeats (SORLA) affects the amyloid precursor protein-dependent stimulation of ERK signaling and adult neurogenesis.. J Biol Chem.

[19] Lafont F, Rouget M, Rousselet A, Valenza C, Prochiantz A (1993). Specific responses of axons and dendrites to cytoskeleton perturbations: an in vitro study.. J Cell Sci.

[20] Lafont F, Rouget M, Triller A, Prochiantz A, Rousselet A (1992). In vitro control of neuronal polarity by glycosaminoglycans.. Development.

[21] Topilko P, Schneider-Maunoury S, Levi G, Trembleau A, Gourdji D (1998). Multiple pituitary and ovarian defects in Krox-24 (NGFI-A, Egr-1)-targeted mice.. Mol Endocrinol.

[22] Prochiantz A, Rousselet A, Chamak B (1990). Adhesion and the in vitro development of axons and dendrites.. Prog Brain Res.

[23] Harada T, Morooka T, Ogawa S, Nishida E (2001). ERK induces p35, a neuron-specific activator of Cdk5, through induction of Egr1.. Nat Cell Biol.

[24] Pignatelli M, Cortes-Canteli M, Santos A, Perez-Castillo A (1999). Involvement of the NGFI-A gene in the differentiation of neuroblastoma cells.. FEBS Lett.

[25] Small DH, Nurcombe V, Moir R, Michaelson S, Monard D (1992). Association and release of the amyloid protein precursor of Alzheimer's disease from chick brain extracellular matrix.. J Neurosci.

[26] Williamson TG, Nurcombe V, Beyreuther K, Masters CL, Small DH (1995). Affinity purification of proteoglycans that bind to the amyloid protein precursor of Alzheimer's disease.. J Neurochem.

[27] Williamson TG, Mok SS, Henry A, Cappai R, Lander AD (1996). Secreted glypican binds to the amyloid precursor protein of Alzheimer's disease (APP) and inhibits APP-induced neurite outgrowth.. J Biol Chem.

[28] Smith-Swintosky VL, Pettigrew LC, Craddock SD, Culwell AR, Rydel RE (1994). Secreted forms of beta-amyloid precursor protein protect against ischemic brain injury.. J Neurochem.

[29] Hoffmann J, Pietrzik CU, Kummer MP, Twiesselmann C, Bauer C (1999). Binding and selective detection of the secretory N-terminal domain of the alzheimer amyloid precursor protein on cell surfaces.. J Histochem Cytochem.

[30] Young-Pearse TL, Chen AC, Chang R, Marquez C, Selkoe DJ (2008). Secreted APP regulates the function of full-length APP in neurite outgrowth through interaction with integrin beta1.. Neural Dev.

[31] Giuffrida ML, Caraci F, Pignataro B, Cataldo S, De Bona P (2009). Beta-amyloid monomers are neuroprotective.. J Neurosci.

[32] Jones MW, Errington ML, French PJ, Fine A, Bliss TV (2001). A requirement for the immediate early gene Zif268 in the expression of late LTP and long-term memories.. Nat Neurosci.

