# Supplementary material for: Secreted Amyloid Precursor Protein β and Secreted Amyloid Precursor Protein α Induce Axon Outgrowth In Vitro through Egr1 Signaling Pathway
Source: PLoS One. 2011 Jan 27;6(1):e16301. doi: 10.1371/journal.pone.0016301 (PMC3029320; doi:10.1371/journal.pone.0016301)
Supplement: Table S1 — Presentations of parameters investigated in Figures 1 , 2 , and 3 . In Figure 1, sAPPs was added to 1DIV neurons at concentrations of 50 to 150 nM; cell adhesion and morphometric analysis was performed 24 hours and 96 hours later, respectively. In Figure 2, sAPP-Fc at 150 nM was added to 5DIV neurons and morphometric analysis was performed 96 hours later. In figure 3, UO126 was added 1 hour before sAPP-Fc at 150 nM on neurons after 1DIV and morphometric analysis was performed 48 hours later. The data are expressed as the Mean ± S.E.M. The levels of significance are indicated by a star and described in the corresponding legends of Figs. 1, 2, and 3. The primary neurons were from mouse embryonic cortex of E16 Swiss strain mice. (DOC) [file pone.0016301.s001.doc]

Table S1. Presentations of parameters investigated in figures 1, 2 and 3

|  |  | Axon length (µm) (Mean ± S.E.M.) | Dendrite length (µm) (Mean ± S.E.M.) | Number of primary neurites per neuron (Mean ± S.E.M.) | Adhesion data (number of neurons per mm²) (Mean ± S.E.M.) |
| --- | --- | --- | --- | --- | --- |
| Figure 1B, C, D, E | Ctrl | 90.0 ± 6.8 | 183.2 ± 11.3 | 7.4 ± 0.3 | 101.7 ± 4.8 |
|  | sAPPα 50 nM | 115.6 ± 7.3 | 82.3 ± 6.1* | 6.6 ± 0.3 | 76.0 ± 3.5* |
|  | sAPPβ 50 nM | 113.7 ± 8.7 | 98.3 ± 8.9* | 6.9 ± 0.3 | 85.0 ± 4.6* |
|  | sAPPα 100 nM | 130.3 ± 8.2* | 75.0 ± 5.4* | 6.2 ± 0.3* | 74.4 ± 2.7* |
|  | sAPPβ 100 nM | 113.5 ± 7.8 | 88.5 ± 7.0* | 6.7 ± 0.4 | 76.7 ± 3.4* |
|  | sAPPα 150 nM | 132.6 ± 9.0* | 148.6 ± 10.4 | 5.7 ± 0.3* | 82.3 ± 3.3* |
|  | sAPP-Fc 150 nM | 127.0 ± 9.2* | 160.5 ± 14.6 | 5.1 ± 0.3* | 76.1 ± 1.5* |
|  | sAPPβ 150 nM | 121.4 ± 7.6* | 113.0 ± 7.9* | 4.8 ± 0.2* | 82.6 ± 3.5* |
| Figure 2 | Ctrl | 102.8 ± 11.5 | 122.9 ± 14.7 | 5.7 ± 0.4 |  |
|  | sAPP-Fc 150 nM | 132.6 ± 10.2* | 104.3 ± 12.7 | 4.7 ± 0.4* |  |
| Figure 3 | Ctrl | 22.6 ± 1.8 | 36.5 ± 2.8 |  |  |
|  | sAPP-Fc 150 nM | 47.4 ± 5.2* | 35.1 ± 3.0 |  |  |
|  | UO126 | 23.1 ± 2.5 | 35.0 ± 2.5 |  |  |
|  | UO126 + sAPP-Fc 150 nM | 30.6 ± 3.5 | 28.5 ± 2.5 |  |  |
