# Supplementary material for: Secreted Amyloid Precursor Protein β and Secreted Amyloid Precursor Protein α Induce Axon Outgrowth In Vitro through Egr1 Signaling Pathway
Source: PLoS One. 2011 Jan 27;6(1):e16301. doi: 10.1371/journal.pone.0016301 (PMC3029320; doi:10.1371/journal.pone.0016301)
Supplement: Table S2 — Presentations of parameters investigated in Figure 5 . In Figure 5, 150 nM sAPP-Fc or sAPPβ were added to 1DIV embryonic cortical neurons from wild type (WT), heterozygous (HT), homozygous (KO) neurons from Egr1 (C57Bl/6J background) mutant mice. Morphometric analysis was performed 96 hours later. The data are expressed as Mean ± S.E.M. The levels of significance are indicated by a star and detailed in the legend of Fig. 5. (DOC) [file pone.0016301.s002.doc]

**Table S2. Presentations of parameters investigated in figure 5**

|  |  | Axon length (µm) (Mean ± S.E.M.) | Dendrite length (µm) (Mean ± S.E.M.) |
| --- | --- | --- | --- |
| Figure 5 | WT ctrl | 82.8 ± 5.9 | 134.8 ± 6.3 |
|  | WT sAPP-Fc 150 nM | 147.3 ± 10.4* | 139.5 ± 6.7 |
|  | WT sAPPβ 150 nM | 137.7 ± 5.1* | 66.4 ± 5.1* |
|  | HT ctrl | 88.4 ± 4.9 | 142.1 ± 6.8 |
|  | HT sAPP-Fc 150 nM | 136.3 ± 9.2* | 129.7 ± 6.3 |
|  | HT sAPPβ 150 nM | 126.5 ± 4.4* | 66.1 ± 4.3* |
|  | KO ctrl | 97.9 ± 6.4 | 139.6 ± 7.1 |
|  | KO sAPP-Fc 150 nM | 121.2 ± 9.3 | 127.6 ± 6.5 |
|  | KO sAPPβ 150 nM | 117.3 ± 4.3 | 73.4 ± 4.3* |
